# Assessment of the quality of groundwater for drinking purposes in the Upper West and Northern regions of Ghana

**DOI:** 10.1186/s40064-016-3676-1

**Published:** 2016-11-22

**Authors:** Sixtus Bieranye Bayaa Martin Saana, Samuel Asiedu Fosu, Godfred Etsey Sebiawu, Napoleon Jackson, Thomas Karikari

**Affiliations:** 1 Department of Dispensing Technology, School of Applied Science and Technology, Wa Polytechnic, Wa, Ghana; 2 Department of Science Laboratory Technology, School of Applied Science and Technology, Wa Polytechnic, Wa, Ghana

## Abstract

**Background:**

Underground water is an important natural resource serving as a reliable source of drinking water for many people worldwide, especially in developing countries. Underground water quality needs to be given a primary research and quality control attention due to possible contamination. This study was therefore designed to determine the physico-chemical and bacteriological quality of borehole water in the Upper West and Northern regions of Ghana.

**Methods:**

The study was conducted in seven districts in Ghana (including six in the Upper West region and one in the Northern region). The bacterial load of the water samples was determined using standard microbiological methods. Physico-chemical properties including pH, total alkalinity, temperature, turbidity, true colour, total dissolved solids (TDS), electrical conductivity, total hardness, calcium hardness, magnesium hardness, total iron, calcium ion, magnesium ion, chloride ion, fluoride ion, aluminium ion, arsenic, ammonium ions, nitrate and nitrite concentrations were determined. The values obtained were compared with the World Health Organization (WHO) standards for drinking water.

**Results:**

The recorded pH, total alkalinity and temperature ranges were 6.14–7.50, 48–240 mg/l and 28.8–32.8 °C, respectively. Furthermore, the mean concentrations of iron, calcium, magnesium, chloride, fluoride, aluminium, arsenic, ammonium, nitrate and nitrite were 0.06, 22.11, 29.84, 13.97, 0.00, 0.00, 0.00, 0.01, 2.09 and 0.26 mg/l, respectively. Turbidity, true colour, TDS and electrical conductivity of the water samples ranged from 0.13 to 105 NTU, 5 to 130 HU, 80.1 to 524 mg/l and 131 to 873 µS/cm, respectively. In addition, the mean total hardness value was found to be 178.07 mg/l whereas calcium hardness and magnesium hardness respectively were 55.28 and 122.79 mg/l. Only 14% of the water samples tested positive for faecal coliforms.

**Conclusion:**

The study revealed that only a few of the values for the bacteriological and physico-chemical parameters of the water samples were above the tolerable limits recommended by the WHO. This calls for regular monitoring and purification of boreholes to ensure good water quality.

## Background

Water is a major constituent of all living things. For example, water makes up approximately two-thirds of the human body weight (Gore 2006). Currently, there are about two billion people worldwide who lack access to safe drinking water (Onda et al. 2012). The consequences of drinking unsafe, contaminated water are numerous and are still not fully understood. According to the World Health Organization (WHO), drinking contaminated water is one of the major causes of diarrheal diseases; these diseases make up the second leading cause of child mortality, resulting in the death of about 760,000 children aged <5 years annually (WHO 2014). For this reason, the WHO has identified the lack of access to clean drinking water as the most critical factor that negatively influences the general health and wellbeing of populations in developing countries (Hoko 2005). Overall, the provision of safe drinking water can help to reduce or eliminate preventable deaths (such as those emanating from waterborne diseases) and improve the quality of life for low-income households around the world (Lawson 2011).

Availability of safe and reliable source of water is an essential prerequisite for sustained population growth and development (Asonye et al. 2007). Groundwater is a vital source of water supply for about one-third of the world’s population (Nickson et al. 2005). For example, over 50% of the water requirements of advanced industrialised countries such as United States of America, Germany and Denmark are derived from groundwater resources (Trauth and Xanthopoulos 1997). According to Boswinkle (2000), groundwater constitutes nearly 90% of the world’s readily available freshwater resources, with the remaining 10% from lakes, reservoirs, rivers and wetlands. In addition, groundwater irrigation of arable lands supports the growth of an estimated 40% of the global agricultural production (DFID 2001). In sub-Saharan Africa, groundwater is the most reliable source of drinking water (Idiata 2011).

Comparatively, groundwater contamination is not as common as surface water but once contaminated, treatment is often difficult and time consuming (Agbaire and Oyibo 2009). Underground water contamination is one of the main environmental issues today due to improper and indiscriminate disposal of sewage, industrial and chemical waste (Obot and Edi 2012). These sources of contamination may influence important biological, physical and chemical variables of groundwater (Sappa et al. 2013). Contaminants that are mainly associated with groundwater pollution include nitrates, pesticides and faecal coliforms. Furthermore, human activities such as land use and the intervention in the natural flow patterns are often implicated in groundwater pollution (Schot and van der Wal 1992). It is therefore critical to first assess the quality of ground water before it can be exploited for human consumption. This is, however, not always the case in many developing countries, sometimes due to financial and poor quality control issues (Hoko 2005). In such countries, physico-chemical and microbiological monitoring of water quality could serve as a convenient tool for examining potential contamination and to help decision-makers in evaluating the effectiveness of regulatory programmes in managing water resources (Pusatli et al. 2009; Song and Kim 2009; Sadiq et al. 2010). These approaches are recognised by the WHO in its *Guidelines for Drinking Water Quality* (WHO 2011). In this document, the WHO outlines its health-based targets for many potential water contaminants. These targets imply any measurable health, water quality or performance variables that are established based on a judgment of safety and risk assessments of waterborne hazards. The health-based targets for contaminants provide a framework for achieving safe drinking water, creating a water safety plan and maintaining water surveillance by policymakers.

Ghana as a lower middle-income country is confronted with various challenges including the provision of potable water for its growing population to meet the Millennium Development Goals and the Sustainable Development Goals (Obuobie and Boubacar 2010). This challenge is even more evident in the northern parts of the country due to the limited number of surface water resources, compelling people to resort to the use of underground water (Sebiawu et al. 2014). These data suggest that groundwater is an important natural resource that affects the health and wellbeing of many people worldwide. Due to this, the quality of this resource should be given a primary research and quality control attention.

Although periodical evaluation of drinking water quality is recommended, this is not the case in many parts of Ghana, particularly in communities that depend solely on boreholes for their water needs (Entsua-Mensah et al. 2007a, b). Owing to this, water contamination events may be missed possibly leading to serious effects on human lives. For example, in a survey of user satisfaction of community water systems in Ghana, respondents expressed concern about possible contamination resulting from equipment corrosion (Entsua-Mensah et al. 2007a, b). Others said that the water turned *milky* after heavy rainfall, suggesting possible contamination by undesirable materials (Entsua-Mensah et al. 2007a, b). The main objective of this study was to evaluate the quality of borehole water in different communities in selected districts of the Upper West and Northern regions of Ghana.

## Methods

### Study area

The study was conducted in six out of the eleven administrative districts in the Upper West region namely Wa West, Wa Municipal, Wa East, Nadowli-Kaleo, Jirapa and Sissala West as well as the Sawla-Tuna-Kalba district in the Northern region of Ghana (Table 1). The districts studied are located in the Guinea Savannah belt of the northwestern corner of Ghana. The climate of the study area follows a general pattern identified within the three regions of northern Ghana (Ghana Districts 2006). It has a single rainy season from April to September, with average annual rainfall of about 115 cm (Ghana Districts 2006). This is followed by a prolonged dry season from early November to March. The mean monthly temperature ranges between 21 and 32 °C. Before the onset of the rainy season, temperatures rise to their maximum (40 °C) and fall to their minimum (20 °C) during harmattan (http://www.ghana.gov.gh/index.php/about-ghana/regions/upper-west). The area has an almost flat topography with elevations generally between 275 and 300 m above sea level (http://www.ghana.gov.gh/index.php/about-ghana/regions/upper-west). The Black Volta river runs through the entire length of the area and it is drained by tributaries, which provide sources of water during the dry season (Cobbina et al. 2012). The study area covers a geographical area of approximately 18,478 square kilometres. Details of the regions, districts and communities from which samples were taken are provided in Table 1. The selection of these communities was motivated by the following reasons:

**Table 1:** Description of study sites and sample collection

Region	District	Community	Number of borehole samples taken	Date of sampling
Upper West region	Nadowli-Kaleo	Nadowli	1	13/4/2015
	Wa West	Bulka, Bulinkyere, Buliyuu, Bungbere, Chabere, Charlie, Daku, Domawaa School, Dornye School, Ga clinic, gurungu, Jagluu Soleyiri, Jinokuraa, Metti, Mwabasi, Nankapaabule, Nanyayiri, Samambo, Siea-Suana, Siira Morazu, Tankara, Wekorbo	22	12/1/2015–16/1/2015
	Wa East	Balazuu, Bayiri, Guonoo, Nahaa, Zinyee Kumasi, Zinye	6	23/12/2014
	Wa Municipal	Islamic SHS, Bamahu, Napogiba Koley, Wa township	5	24/1/2015
	Jirapa	Jirapa Farms, Jirapa Township, Jirapa Township, Hiineteng N-Nyarateng	4	14/2/2015–10/4/2015
	Sissala West	Fielmuo, Suleteng, Gyanvuur, Liero, Zienle’s house, Liero Nyokubagr	5	24/7/2014–15/4/2015
Northern region	Sawla-Tuna-Kalba	Dandanpiri, Dankomplayiri, Nochiteyiri, Sagbayiri, Sey-Goyiri, Tuna-Balie, Wula	7	1/4/2015

1. Rural communities and small towns are not served by central water production and treatment systems that carry out regular quality control checks.
2. Regular quality analysis is not conducted on the boreholes that serve these communities.
3. The borehole water often takes its source from streams and rivers that may become contaminated with solid waste, chemicals and faeces.

### Geological framework

The geology of the study area is characterised by basement crystalline rocks derived from the Precambrian era and principally comprise the Birimian rocks and associated granitoid intrusions (Leube et al. 1990; Taylor et al. 1992; Hirdes et al. 1992). The Birimian rocks include biotite and muscovite—bearing granite, granodiorite, diorite and gabbro, phyllites, schist, tuffs, basalt, sandstones, siltstones and strongly deformed metamorphic rocks (Nude and Arhin 2009). These rocks occur in the southwest and the northeast of the study area whilst Upper Birimian rocks comprising metamorphosed lavas and pyroclastic rocks underlie the south-eastern portion of the area. Basal sandstone of the Lower Voltaian System also underlies the extreme northeast section of the study area.

The basement crystalline rocks are essentially impermeable with very little porosity. The existence of ground water is as a result of chemical weathering and fracturing leading to the formation of aquifers (Obuobie and Boubacar 2010). The depth of the aquifers in the northern part of Ghana are semi confined and have depth ranging from 10 to 60 m. The aquifer recharge has been found to be mainly through precipitation (Obuobie 2008; Martin 2006).

### Sample collection

A total of 50 samples from boreholes were collected between July 2014 and April 2015 for the study. The nozzle of each borehole was flamed with a lit cotton wool soaked in 98% alcohol for about 2 min to achieve sterility. The borehole water samples were aseptically collected from the source using sterilised glass bottles after pumping out water for about 2 min to flush out stagnant water in the pipes. All the water samples were collected into 500 ml sterilised plastic bottles. The bottles were sterilised by washing with 5% nitric acid, and then rinsing several times with distilled water. This was carried out to ensure that the sampling bottles were free from all forms of contaminants. The samples were then kept in an ice container and transferred to the water quality analysis laboratory at the Upper West regional office of the Ghana Water Company in Wa for analysis.

### Physico-chemical analysis

The water temperature, pH, conductivity and total dissolved solids were measured at the point of sampling using handheld multipurpose meters (370 and 470 Jenway, Staffordshire, UK). The total alkalinity on the other hand was determined by titrating each water sample with 0.02 M H_2_SO_4_ using methyl orange as the indicator (American Public Health Association 2005). Turbidity of each water sample was also determined using a turbidity meter (HANNA HI 93703, HANNA Instruments, TX, USA). Total iron concentration, total hardness and nitrate/nitrite concentration were determined using Aquachek^®^ Iron (Hach, USA), Aquachek^®^ Total Hardness (Hach, USA) and Aquachek^®^ Nitrate/Nitrite (Hach, USA) test strips respectively according to the manufacturer’s directions. Calcium ion concentration and calcium hardness were measured by ethylenediaminetetraacetic acid titrimetric method using powdered ammonium murixide as an indicator. The magnesium hardness was calculated by subtracting the value for the calcium hardness from the value for the total hardness. The magnesium ion concentration was then determined by multiplying the value of the magnesium hardness by a constant factor of 0.243 as suggested by American Public Health Association (2005). Chloride ion concentration was determined by titrating each sample against 14 mM silver nitrate solution using 1 ml of 5% potassium chromate as an indicator. Fluoride ion concentration was determined by employing the Spadn’s colorimetric method. UV/VIS spectrophotometer (DR 2400, Hach, Germany) was used to determine the ionic concentrations of arsenic, aluminium and ammonia. Standard solutions were prepared for each metal using suitable metals of each element to be determined. The required wavelength for each metal was used: arsenic at 193.7 nm, aluminium 584 nm and ammonia 425 nm.

### Determination of total and faecal coliforms

The Most Probable Number (MPN) was used to determine the presence of faecal coliforms in the ground water samples. Five double strength MacConkey broth tubes containing inverted Durham tubes were inoculated with 10 ml of water sample; five single strength broth tubes were inoculated with 1 ml of the water; another set of five single strength broth tubes were inoculated with 0.1 ml of the same sample. After incubation for 48 h the number of tubes showing positive colour change, growth and gas production in each set was compared with the MPN index to determine the number of coliforms per 100 ml (American Public Health Association 2005). Furthermore, positive MacConkey broth tubes from the highest dilutions were inoculated into Eosin-Methylene blue agar, brilliant green lactose bile broth and gram stained to confirm the presence of faecal coliforms.

### Ground water quality analysis

Seventeen (17) parameters were considered in calculating the WQI. As shown in Table 2, each of the chemical parameters was assigned a weight (wi) based on its perceived effect on primary health or the relative importance of the chemicals in the overall quality of water for drinking purposes (Vasanthavigar et al. 2010). The highest weight of 5 was assigned to parameters that have major effects on water quality and their importance in quality and a minimum of 1 was assigned to parameters that were considered as not harmful to human health (Table 2).

**Table 2:** Assignment of weights in the ground water WQI

Parameter	Weight (wi)	Relative weight (W_i_)	WHO standards
pH	3	0.05	8.5
Conductivity	3	0.05	1400
Total dissolved solutes	5	0.09	1000
Temperature	1	0.02	25
True colour/appearance	1	0.02	15
Turbidity	1	0.02	5
Total Hardness	3	0.05	500
Calcium ions	3	0.05	75
Magnesium ions	3	0.05	50
Chloride ions	5	0.09	250
Nitrate-nitrogen	5	0.09	10
Nitrite-nitrogen	4	0.07	3
Total iron	1	0.02	0.3
Ammonium ions	5	0.09	1.5
Fluoride	5	0.09	1.5
Aluminium	5	0.09	0.2
Arsenic	5	0.09	0.01
	∑wi = 58	∑W_i_ = 1.00	

*WHO* World Health Organization

The method used to determine the WQI was first proposed by Tiwari and Mishra (1985) and later modified by Dhakad et al. (2008) on groundwater samples which involves first calculating the quality of parameters, **qi** as presented in the equation below

1 $${\textbf{qi}} = \frac{Va}{Vs} \times 100$$

where **qi** is the quality rating of each parameter for a total of *n* water samples, **Va** is the value of the water-quality parameter obtained from the laboratory analysis of the sample and **Vs** is the value of the water-quality parameter obtained from the WHO water-quality standard.

The relative weight **W**_**i**_ was also calculated from the following equation:

2 $${\text{W}}_{\text{i}} = \frac{wi}{\sum wi}\quad {\text{where}}\;wi\;{\text{is}}\;{\text{the}}\;{\text{weight}}\;{\text{of}}\;{\text{each}}\;{\text{parameter}}.$$

Finally, the WQI was then determined by summing the product of the quality rating of each parameter and its relative weight.

3 $${\text{WQI }} = \sum \left( {qiWi} \right)$$

The WQI obtained was afterwards used to determine the water quality of each sample according to Table 3 [prepared according to data from Vasanthavigar et al. (2010)].

**Table 3:** Classification of water quality based on the WQI

WQI range	Type of water
<50	Excellent water
50–100.1	Good water
100.1–200.1	Poor water
200.1–300.1	Very poor water
>300	Water unsuitable for drinking

## Results and discussion

The quality of drinking water is a major determinant of health for users. For this reason, periodic quality control measures are necessary. However, groundwater sources in many rural communities in Ghana lack regular quality control checks. This study was therefore aimed at evaluating borehole water quality in selected communities in northern Ghana, with a view to provide data that might be useful to policymakers and water service providers. It was identified through our physico-chemical and bacteriological quality analysis that most of the water samples were within the acceptable quality parameters recommended by the WHO. However, 14% of the samples tested positive for faecal contamination, emphasising the importance of regular quality assessment measures by the responsible agencies.

The physico-chemical parameters for all the water samples analysed were within the standards of the WHO except temperature, turbidity, true colour and magnesium ion concentration (WHO 2011). The pH of all the water samples were within the range of 6.14–7.50 indicating that the water was slightly acidic to neutral with a mean value of 6.87 ± 0.13. In fact, the geology of the sampling site could partly contribute to the final pH of the ground water. According to Pelig-Ba (1998), the geology around the aquifers of the study area is dominated by crystalline silicate rocks and regolith which impart acidity to the water. The pH values recorded in this study are similar to that of a previous study by Sebiawu et al. (2014) which reported an average pH of 6.57. However, our water pH data differed slightly from an investigation into physico-chemical properties of groundwater for irrigation purposes in the Upper West region conducted by Salifu et al. (2015), which reported a mean pH of 7.30. This slight pH variation can be explained by the difference in study sites used for the two studies. Yet, the closeness of the mean pH value reported herein to that reported by the two earlier studies suggests that groundwater in the study area is slightly acidic to neutral.

The value of electrical conductivity depends on the concentration, types of soluble ions as well as the temperature of the water (Hem 1985). In this study, the conductivity values ranged between 131 and 873 μS/cm (Table 4). The mean conductivity value for all samples analysed was 373.32 μS/cm with a standard deviation of 175.49 μS/cm (Table 4). It was observed that, the conductivity of the water samples was influenced by TDS (r = 0.99), Ca^2+^ (r = 0.72) and total hardness (r = 0.56) (Table 6). Generally, groundwater in this study had low electrical conductivities, implying low mineral content and may therefore be referred to as fresh water.

**Table 4:** Physico-chemical parameters of borehole water samples from selected communities in the Upper West and Northern regions of Ghana

	Mean	Median	SD	Minimum	Maximum	WHO standard	Percentage compliance (%)
pH	6.87	6.87	0.31	6.14	7.50	8.50	100
Conductivity	373.32	320.50	175.49	131.00	873.00	1400.00	100
TDS	218.21	192.70	109.20	80.10	524.00	1000.00	100
Temperature	30.88	31.00	0.90	28.80	32.80	25.00	0
True colour	11.46	5.00	22.26	5.00	130.00	15.00	94
Turbidity	4.49	0.63	15.29	0.13	105.00	5.00	92
Total alkalinity	121.82	112.50	48.73	48.00	240.00	ND	ND
Total hardness	178.07	185.00	100.62	22.00	337.50	500.00	100
Ca hardness	55.28	51.00	28.24	14.00	116.00	ND	ND
Mg hardness	122.79	121.50	84.87	6.00	311.50	ND	ND
Calcium ions	22.11	20.40	11.30	5.60	46.40	75.00	100
Magnesium ions	29.84	29.50	20.62	1.46	75.70	50.00	78
Chloride ions	13.97	15.00	6.36	5.30	44.00	250.00	100
Nitrate-nitrogen	2.09	2.00	1.33	0.00	6.00	10.00	100
Nitrite-nitrogen	0.26	0.05	0.39	0.00	2.00	3.00	100
Total iron	0.06	0.04	0.05	0.00	0.25	0.30	100
Ammonium ions	0.01	0.00	0.02	0.00	0.08	1.50	100
Fluoride ions	0.00	0.00	0.00	0.00	0.00	1.50	100
Aluminium ions	0.00	0.00	0.00	0.00	0.01	0.20	100
Arsenic	0.00	0.00	0.00	0.00	0.00	0.01	100
Most probable number (MPN) index	1.60	0.00	4.48	0.00	16.00	0.00	86

Total dissolved solids (TDS) in drinking water has been associated with natural source, sewage, industrial wastewater, urban run-off and chemicals used in water treatment process (Environmental Protection Agency, Ghana 2002). High concentrations of TDS may confer undesirable taste, odour and colour on water, posing adverse reactions to the consumer (Spellman and Drinan 2012). The TDS in this study exhibited a wide variation with a minimum value of 80.1 mg/l and a maximum value of 524 mg/l (Table 4). All the TDS values were below the maximum allowable value of 1000 mg/l prescribed by the WHO (2011). Moreover, these results are similar to those obtained by Salifu et al. (2015) and Sebiawu et al. (2014) which showed that the average TDS of underground water in the Upper West and Northern regions averaged 200 mg/l.

High potable water temperature may impart undesirable taste and odour as well as the corrosive ability of the water (WHO 2011). This may also facilitate the growth of microorganisms, hence affecting water quality (WHO 2011). In this study, sample temperatures were between 28.8 and 32.8 °C (Table 4). These temperatures were all above the WHO maximum limit of 25 °C. This could be attributed to the environmental temperature as well as other climatic conditions prevailing in the study area at the time of sampling. Northern Ghana, due to its tropical savannah features, records higher temperatures compared to other parts of the country. Hence, the water temperatures recorded here may only highlight environmental characteristics without any suggestion for adverse effects on human health.

The true colour of all the water samples were within the acceptable limits prescribed by the WHO, except four samples whose appearance was above the maximum limit of 15 HU (Table 4). The water samples whose colours were outside the WHO standards were from Chabere, Guonoo, Nahaa and Suleteng communities. The water samples from these communities presented a milky colour which may be due to the presence of particulate matter arising from underground clay and other rock fragments surrounding the water source (WHO 2004). Flooding of areas around boreholes has also been found to deposit silt and clay into boreholes, thereby affecting the colour of borehole water (McMahon 2010). The colour of water samples from Chabere and Suleteng communities pointed to a possible contamination from surface water runoffs since water from these communities were also found to contain faecal coliforms. The mean true colour of the samples investigated was 11.46 ± 22.26 HU (Table 4).

Turbidity of the water samples showed wide variations ranging from 0.13 NTU to as high as 105 NTU (Table 4). The turbidity values of all the samples were within the maximum acceptable limits of the WHO standard except samples from Chabere, Gounoo, Nahaa and Suleteng whose turbidity were 17, 10, 29 and 105 NTU respectively. The milky nature of water from these communities may result from possible underground clay contamination.

Alkalinity is the acid neutralising ability of the water (United States Environmental Protection Agency 2012). Alkalinity of water is mainly caused by the presence of ions such as HCO_3_^−^, CO_3_^2−^ or OH^−^ in ground water (United States Environmental Protection Agency 2012). We identified that alkalinity of the water samples was fairly low and within the WHO standard (Table 4) with a mean value of 121.82 mg/l ± 48.73. The low alkalinity of the water samples in the study area may be connected to the geology of the area which is dominated by crystalline silicate rocks and weathered derivatives (regolith). These rocks have been identified to impart acidity to underground water (Obuobie and Boubacar 2010).

Total hardness is chemically expressed as the total concentration of Ca^2+^ and Mg^2+^ as milligram per liter equivalent of CaCO_3_ (Nitsch et al. 2000). Physically, hardness could be referred to as the resistance of water to lather soap (Todd 2008). The total hardness values recorded ranged from 22 to 337.5 mg/l for all the samples analysed (Table 4). The total hardness measurements for all the samples were below the 500 mg/l recommended by the WHO for drinking water (Table 4), suggesting that they were all compliant with the WHO guideline and also safe for drinking.

The average calcium ion concentration for the borehole water samples was 22.11 mg/l providing a hardness of 55.28 mg/l (Table 4). Calcium ion (Ca^2+^) can occur naturally in ground water through the dissolution of carbonate minerals and the decomposition of sulphate, phosphate and silicate minerals (Cobbina et al. 2012). The low concentrations of Ca^2+^ observed in this study could be attributed to the absence of sulphate and phosphate containing rocks in the study area rather than pollutants (Nude and Arhin 2009).

The magnesium ion (Mg^2+^) concentration in all samples was lower than the WHO standard of 50 mg/l for potable water (Table 4). The mean concentration of Mg^2+^ was 29.84 ± 20.62 mg/l. The main sources of magnesium in the underground water sampled may be attributed to geological sources such as dolomite, biotite and pyroxenes (Fetter 2000) are abundant in the basement rocks of the sampled area (Key 1992). The Mg^2+^ concentrations observed here are in agreement with previous reports by Cobbina et al. (2012), Sebiawu et al. (2014) and Salifu et al. (2015).

Ammonium as a form of nitrogen is also found in groundwater, primarily from the discharge of wastewater from sources such as septic systems and wastewater infiltration beds (Böhlke et al. 2006). Some of the water samples (24%) were found to contain ammonium ions although at low concentrations (mean = 0.01 ± 0.02 mg/l; Table 4) as compared to the WHO maximum acceptable limit of 1.5 mg/l, suggesting that drinking the borehole water analysed carried little risk of ammonia-related negative impacts on human health.

Excess chloride ions in water may not pose any health risk to consumers; however, high concentrations of chloride and sodium ions in water may interact to form sodium chloride which could impart a salty taste to the water (Cobbina et al. 2012). Chloride content (mean = 13.97 ± 6.36 mg/l) was lower than the maximum acceptable limit of 250 mg/l recommended by the WHO. Thus, the concentration of the chloride was considered satisfactory.

Nitrate and nitrite are the forms of nitrogen most commonly associated with groundwater contamination (Böhlke et al. 2006). Although the presence of nitrate does not pose any health threat to adults, ingestion by infants can cause low oxygen levels in the blood, a potentially fatal condition (Spalding and Exner 1993). For this reason, the WHO has established a drinking-water maximum allowable threshold of 10 mg/l nitrate as nitrogen (WHO 2011). All borehole samples analysed contained varying concentrations of nitrate ranging from 0.0 to 6 mg/l with mean and standard deviation of 2.09 and 1.33 mg/l, respectively (Table 4). This low concentration of nitrate observed in this study is in agreement with the findings of Mueller et al. (1995), which reported nitrate concentrations in underground water to be less than 2 mg/ml. The concentration of nitrate in the water samples was within the permissible range of 0.0–10.0 mg/l as recommended by the WHO for drinking water (Table 4). Also, the concentration of nitrite in the water samples varied between 0.0 and 2 mg/l with a mean value of 0.26 mg/l. Nitrite and nitrate concentrations of the samples of borehole water were within the permissible limits of 0.0–3.0 mg/l in line with WHO standards for drinking water (Table 4).

High concentration of iron in groundwater may not pose any health hazards but may not be patronised by consumers due to unpleasant odour and taste that is normally associated with underground water with higher iron concentrations (Gardner and Pelig-Ba 1995). Iron in groundwater may be derived from natural sources as well as steel pumps and casings (Sebiawu et al. 2014). The total iron concentration was between 0.0 and 0.25 mg/l with mean value of 0.06 ± 0.05 mg/l (Table 4). The total concentration of iron was within the range of 0.0–0.3 mg/l as per the WHO standard. Thus, the water from all the samples is considered satisfactory for human consumption. From our study, there was negative correlation between iron concentration and pH (r = −0.29) even though low pH may cause an increase in iron content derived from the rocks surrounding the aquifers (Pelig-Ba 1998).

All samples collected had no detectable concentrations of fluoride, aluminium and arsenic. Regions where the bedrock composition is dominated by granite tend to experience high fluoride concentration problems (Smedley et al. 1995). However, the results from our study showed no detectable concentrations of fluoride even though the geology of the study area is dominated by granite. Also, areas most likely to have elevated concentrations of aluminium are those where the ground waters are acidic, particularly from the Birimian province where this study was undertaken (Pelig-Ba 1998). The results, however showed negligible concentrations of aluminium. This finding suggests no immediate threat to the health of individuals in the study area who drink this water. These results corroborate that of Salifu et al. (2015) but contradict that of Cobbina et al. (2012) which found traces of arsenic (0.033 mg/l) and fluoride (2.9 mg/l) in ground water samples in the Sawla-Tuna-Kalba district of the Northern region of Ghana.

A wide range of microorganisms can be present in drinking water and it is often impossible to test for all of these microorganisms (WHO 2011). In order to monitor microbial quality of drinking water, certain indicator microorganisms can be measured to test for faecal pollution. Here, we used the MPN assay to test for the presence of common bacterial contaminants in the borehole water samples. Overall, 14% of groundwater samples tested exceeded the recommended 0.0 cfu/100 ml for coliforms by the WHO, indicating the presence of bacterial contaminants. The total number of coliforms ranged from 0.0 cfu/100 ml to more than 16 cfu/100 ml with a mean of 1.60 cfu/100 ml (Table 4). The presence of coliforms in these groundwater samples studied can be attributed to the absence of residual chlorine, an indication that the borehole water had not been chlorinated prior to sampling. Presence of coliforms in infected boreholes renders the water unwholesome, unless it is chlorinated. This is because excess coliforms may give an indication of the presence of other pathogens, which could cause waterborne diseases such as typhoid fever, hepatitis, gastroenteritis and dysentery (Lawson 2011).

The WQI datasets resulting from the 50 samples ranged from 10.81 to 55.87 and were categorised accordingly as being excellent water, good water, poor water and very poor water (Table 5). Majority of the water samples 49 (98%) were classified as “excellent water” whereas only 1 (2%) was classified as “good water”. None of the water samples had WQI within the categories of “poor water” and “very poor water”. Therefore, all the water samples without the presence of coliforms were considered suitable for human drinking. Since WQI is dependent on physico-chemical parameters, the values of WQI making the water suitable for drinking can be inferred from the acceptable concentration of parameters such as TDS, turbidity, conductivity, nitrates, nitrites, total hardness, calcium ions and magnesium ions. In order to evaluate potential correlations between the groundwater physico-chemical properties studied, the Spearman’s correlation co-efficient was used. Particularly, there were significant positive correlations between WQI and the physico-chemical parameters listed above, further confirming the relation between WQI and these parameters (Table 6).

**Table 5:** Classification of water samples analysed based on the WQI

WQI values	Interpretation	Number of samples (%)
<50	Excellent water	49 (98%)
50–100.1	Good water	1 (2%)
100.1–200.1	Poor water	0 (0%)
200.1–300.1	Very poor water	0 (0%)

**Table 6:** Spearman’s correlation co-efficients for the physico-chemical parameters and WQI for borehole water samples from selected communities in the Upper West and Northern regions of Ghana

	PH	Cond	TDS	Temp	TC	Turb	Total Hardness	Ca	Mg	Cl	Nitrate	Nitrite	Fe	NH_4_^−^	Al	WQI
pH	1.00															
Conductivity (cond)	0.39	1.00														
TDS	0.34	*0.99*	1.00													
Temperature	0.22	0.00	−0.02	1.00												
True colour (TC)	−0.10	0.25	0.25	−0.02	1.00											
Turbidity (Turb)	0.16	−0.11	−0.12	−0.01	0.31	1.00										
Total Hardness	−0.07	*0.56*	*0.62*	−0.17	0.16	−0.10	1.00									
Ca	0.25	*0.72*	*0.73*	0.01	0.28	0.05	*0.65*	1.00								
Mg	−0.17	0.43	0.49	−0.21	0.09	−0.14	*0.97*	0.44	1.00							
Cl	0.06	0.27	0.24	0.01	0.22	−0.13	−0.06	0.10	−0.10	1.00						
Nitrate	−0.12	0.43	0.49	−0.05	0.31	−0.14	*0.59*	0.36	*0.58*	0.16	1.00					
Nitrite	−0.15	0.43	0.46	0.00	0.49	0.01	*0.54*	0.40	*0.51*	0.26	*0.74*	1.00				
Total Iron	−0.29	0.01	0.05	−0.05	0.09	−0.07	0.28	0.13	0.29	0.02	0.39	*0.51*	1.00			
NH_4_^−^	0.24	−0.06	−0.06	0.06	−0.07	−0.06	−0.13	−0.24	−0.08	0.14	0.04	−0.17	−0.17	1.00		
Al	−0.19	−0.18	−0.17	−0.41	−0.08	−0.08	−0.21	−0.30	−0.14	0.11	−0.12	−0.05	−0.02	−0.07	1.00	
WQI	0.05	*0.53*	*0.57*	−0.10	*0.61*	*0.52*	*0.70*	*0.61*	*0.63*	0.06	*0.57*	*0.66*	0.25	−0.12	−0.20	1.00

Analysis was carried out at p ≤ 0.05. Numbers in italics indicate significant positive correlations

## Conclusion

From a water quality point of view, most of the data for the physico-chemical parameters indicated tolerable quality. Bacteriological quality of a few of the water samples analysed in this study did not meet the standard requirements set for drinking water by the WHO. Thus, the heavy reliance on borehole water in the study area calls for constant monitoring and design of regular purification strategies by government agencies concerned to ensure good water quality.

## Abbreviations

TDS: total dissolved solids
WQI: water quality index
WHO: World Health Organization
